# Caring for data: Value creation in a data-intensive research laboratory

**DOI:** 10.1177/0306312720906567

**Published:** 2020-02-13

**Authors:** Clémence Pinel, Barbara Prainsack, Christopher McKevitt

**Affiliations:** Centre for Medical Science and Technology Studies, Department of Public Health, University of Copenhagen, Denmark; Department of Political Science, University of Vienna, Austria; Department of Global Health & Social Medicine, King’s College London, UK; School of Population Health & Environmental Sciences, King’s College London, UK

**Keywords:** care, data, data-intensive research, ‘omics’ research, relational ontology, valuation

## Abstract

Drawing upon ethnographic observations of staff working within a research laboratory built around research and clinical data from twins, this article analyzes practices underlying the production and maintenance of a research database. While critical data studies have discussed different forms of ‘data work’ through which data are produced and turned into effective research resources, in this paper we foreground a specific form of data work, namely the affective and attentive relationships that humans build with data. Building on STS and feminist scholarship that highlights the importance of care in scientific work, we capture this specific form of data work as care. Treating data as relational entities, we discuss a set of caring practices that staff employ to produce and maintain their data, as well as the hierarchical and institutional arrangements within which these caring practices take place. We show that through acts of caring, that is, through affective and attentive engagements, researchers build long-term relationships with the data they help produce, and feel responsible for its flourishing and growth. At the same time, these practices of care – which we found to be gendered and valued differently from other practices within formal and informal reward systems – help to make data valuable for the institution. In this manner, care for data is an important practice of valuation and valorisation within data-intensive research that has so far received little explicit attention in scholarship and professional research practice.

## Introduction

**Figure 1.**
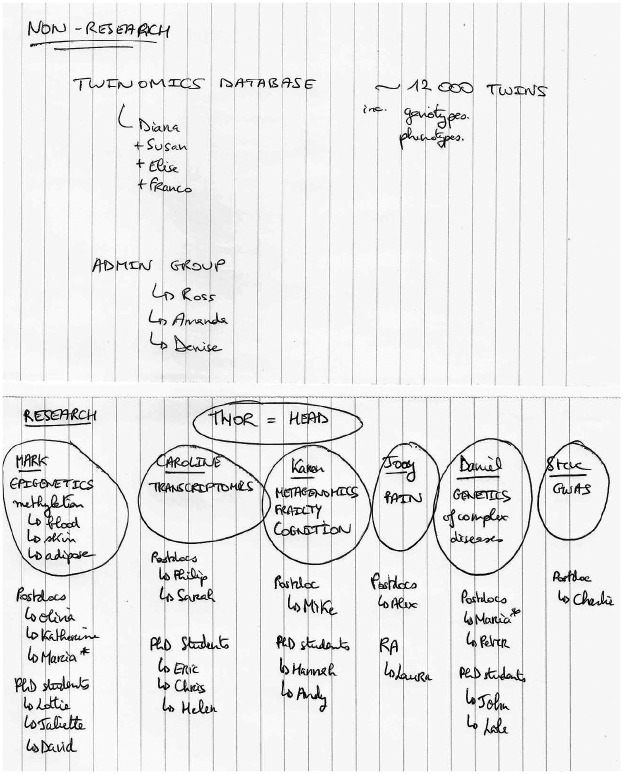
Organizational chart at Twinomics (copied from the original version, using pseudonyms).

The walk to the laboratory takes me through the local hospital. Today is my first day at Twinomics, a genetics research laboratory focused on the study of twins. Holding a large box of fresh home-made madeleines, a gift that I will present to the lab members, I make my way up to the laboratory. At the reception, I meet Olivia, a postdoc in the lab who has been appointed as my ‘sponsor’ for the duration of fieldwork at Twinomics. She takes me on a tour of the lab. We start with the admin room where, she explains, ‘non-research staff’ work on building and maintaining the twins database. They include, for example, research nurses collecting samples from twins in clinical visits and research assistants entering data onto the database. This is a large space with, on one side, staff sitting at their desks, some working on their computers, others talking on the phone, and on the other side, an archive room where the lab holds records of the twins in the database. As we walk past that room, I take a brief look and see rows of tall shelves reaching the ceiling, packed with folders. Olivia, pointing to the shelves, says ‘Yeah, it’s a bit messy’, and laughs. We continue our tour and enter the analysts’ room. The analysts are post-doctoral researchers working across the different teams of Twinomics. They conduct analyses of the data held in the twins database to make new claims about the world. There is a stark contrast between the atmosphere in the admin room, which is bustling with people chatting with one another, and where telephones are ringing, and the quiet of the analysts’ room. The room is organized in four rows of desks. Each desk is set up with a computer and one or multiple screens. Olivia points to her desk, and then to another one that I learn has been allocated to me. I put down the box of madeleines and offer one to Olivia. We sit down by the desk, Olivia grabs a madeleine and we start talking about my stay at Twinomics. To answer my questions about the lab and describe best ‘how [they] work’ Olivia takes my notebook and starts drawing. She explains that each team is organized around an ‘omic’ dataset. Her team, the epigenetics team, works on the DNA methylation datasets. The work of the lab revolves around several main research areas: epigenetics, microbiome, transcriptomics and ‘pain-omics’. The Twinomics’ research portfolio is not organized around the study of a specific disease, but it is held together by its twins database. Work at the lab is centred around an assemblage of data gathered over the past 20 years. Olivia draws two diagrams: one titled ‘Research’, structured around the general principle of ‘a team per dataset’ and a Principal Investigator (PI) leading each team, and another titled ‘Non-research’ composed of staff supporting the lab’s research by building and maintaining the twins database (see Figure 1 below). The twins data is what brings together staff at Twinomics, and as we will show, every member of the lab is involved in producing, maintaining and making data valuable.

The above is an extract from fieldnotes written by the first author (CP) during her ethnographic fieldwork. Names and identifying details of places and individuals have been changed. We begin with this scene from the first day of fieldwork at Twinomics because it illustrates how data exist and are made valuable within specific knowledge infrastructures made of people, institutions and relationships.

In this paper, drawing on the case of Twinomics, we discuss the human labor underlying the production and maintenance of data. We show that data result from deeply relational practices enabled by human interactions, affection and attention, bodies, expertise, tacit knowledge, tools and technologies. We specifically put to the foreground how data are ‘cared for’ by staff. As staff mobilize time, knowledge and affect to make data, we argue that they form caring relationships with the data they help create. We outline what these caring practices help accomplish within Twinomics, pointing out how they shape both the data, transforming it into a valuable research resource essential for the production of reliable results, and the researchers, in that it creates a lasting link between researchers and data, which then lead to further investments of time and affect by researchers. As such, data caring practices represent an important mechanism of value creation for the laboratory.

Postgenomic research in the 21^st^ century is often described as data-driven or data-centric (Kell and Oliver, 2004; Leonelli, 2012; Meloni, 2016; for other ways of understanding postgenomics see Richardson and Stevens, 2015). That is, it is thought to be led by the generation, collection and potential interpretation of vast quantities of data in order to identify new processes and phenomena. Within the biomedical sciences, data from diagnostic tests, medical records, digital devices or ‘omic’ sciences (sequencing data) are being ‘resourced’ (Hoeyer et al., 2017) through a series of practices aimed at producing, collecting, curating and storing data, while it is made available to various actors for a number of purposes, from research to governance or economic growth. Some commentators speak of a ‘data deluge’ (Economist, 2010; *Nature Biotechnology*, 2008) produced by sophisticated high-throughput technologies such as next-generation sequencing or micro-array experiments, without sufficient knowledge and tools being available to make sense of these data (Fiske et al., 2019; Neff, 2013).

As part of the Big Data rhetoric articulated by media outlets or tech companies, data are often treated as objective ‘givens’ which are ‘out there’ like natural resources such as water or oil (e.g. The Economist, 2017; Puschmann and Burgess, 2014). Data are seen as neutral entities that represent the social and natural world and that exist independently of those who collect and analyze them. Critical data studies have provided more nuanced accounts of data-intensive healthcare and research by, for example, exploring the role of data in contemporary science and interrogating how it is generated and mobilized for valuable endeavors (e.g. Leonelli, 2016; Neff et al., 2017), or studying the effects of new digital technologies such as self-tracking apps on patients or doctors (e.g. Lupton, 2017). Authors have questioned the revolutionary character of data-driven science (Müller-Wille and Charmantier, 2012; Strasser, 2012), pointing to the historical continuities between contemporary and older forms of biological research, while they have also paid attention to the epistemology, expectations and effects of Big Data in biomedical sciences (Kitchin, 2014; Leonelli and Ankeny, 2012; Tanweer et al., 2016). Scholars have analyzed the assumptions and values embedded in data, underlining the contextual and relational nature of data (Neff et al., 2017; Prainsack, 2019). This points to the human work that goes into so-called data-driven research, suggesting that data are never ‘raw’ (Gitelman, 2013) nor neutral, but are embedded in relationships with the people and instruments that help create and maintain them. Authors have pointed to different forms of ‘data work’ necessary to produce data, shedding light, for example, on the ways researchers transform data to make it ‘open’ in government open data programs (Denis and Goëta, 2017), or the invisible and informal processes through which staff produce metadata (Edwards et al., 2011; Mayernik, 2019). Leonelli’s work on large-scale databases has helped to demystify the role of technology in data-intensive biology, demonstrating the material, social and institutional circumstances by which data are made available and ready to use in knowledge production. In a series of influential publications (Ankeny and Leonelli, 2015; Leonelli, 2010, 2016; Leonelli and Ankeny, 2012), she has pointed to a set of practices undertaken by dozens, if not hundreds of people, to handle and work with data within large-scale databases. She has focused in particular on the work of data curators, or ‘packaging experts’, who select, prepare and classify data to make it available in databases, thus enabling its circulation and re-use within new research contexts. Data curation, Leonelli underlines, requires considerable skill and expertise at the bench to be able to recognize what later data users will require as information about the origin of data, but also familiarity with several fields of biological research to be able to annotate and label data.

In this critical body of work, rather than being understood as finite objects and neutral evidence about the social and material world, data are seen as emergent and relational entities, crafted by human and non-human actors and in relation to particular historical and social circumstances. In particular, authors draw attention to the multiple human practices, labor and investments that are involved in making data. In this article, we explore a specific dimension of data work, which has been under-attended to in existing scholarship, namely the personal relationships humans tie with data as they invest time, knowledge and affects in the process of producing it. We conceptualize this form of data work as care. While this focus on care is relatively new to critical data studies, in other bodies of scholarship, ‘matters of care’ (de la Bellacasa, 2011) are important areas of interest. Feminist scholarship has pointed to the often-devalued, yet constitutive, forms of care in scientific work (e.g. Friese, 2013; Latimer and Miele, 2013; Martin et al., 2015). Bringing this critical scholarship on caring practices into dialogue with analysis of data curation and other data practices has, we argue, a number of advantages. First, it helps to shed light on the invisible human labor of data-intensive research, specifically pointing to and problematizing the hierarchical and gendered arrangements within which data practices take place. Second, it puts to the foreground the relational, affective and attentive dimensions of data work, and helps us question the constitutive nature of these specific data practices. Finally, it can elucidate an important aspect in and through which research data obtain value, namely through the affective and attentive relationships that staff build with the data that they produce or take care of. Understood in this way, data care is a specific type of data work, rather than a class of activities that is separate from others. Most of the data care practices we describe also contain elements of other investments and engagements, including strategic or instrumental ones. Foregrounding the affective, attentive and relational dimensions of data work – enshrined in our notion of data care – enables us to see things that we would not otherwise see.

In what follows, we first outline the analytical framework employed for this study. We provide a synthesis of the critical scholarship on care, underlining what the notion of care can enable us to do when thinking about data-intensive research. We then discuss our research methods and briefly describe the laboratory in which the study was carried out. This is followed by an analysis of researchers’ care practices in the laboratory. While our case study is concerned with genomic research data, we believe that the observations we make in this field are relevant to other contexts where research data are created.

## The affective and relational dimensions of data work

Scholarship on care cuts across a range of literatures in the humanities and social sciences. Proponents of the care ethics approach understand care as a set of practices that seek to foster, preserve and repair our world by maintaining a network of social relations (Fisher and Tronto, 1990; Ruddick, 1989; Tronto, 1994). Within this tradition, we distinguish between two main approaches depending on how the relationship between the giver and the receiver of care is conceived of. In the first approach, which we call the ‘transaction’ approach, the giver and receiver of care are seen to be independent entities where one ‘chooses’ to care for another (Engster, 2007; Noddings, 1982). This approach is influenced by the pronounced history of atomistic individualism in North American thought especially (Siedentop, 2014). The second approach, which we call the ‘mutual constitution’ approach, is underpinned by a strong relational ontology (Mackenzie and Stoljar, 2000; Nedelsky, 2011; Taylor, 1989). In this approach, givers and receivers of care are not independent entities, but they are connected through joint practices, environments and experiences, to such an extent that they become mutually constitutive of each other′s interests and subjectivities. Care is not a ‘choice’ of an independent actor, but a practice that partly emerges from, and in turn strengthens, the connectedness between entities (Held, 1993, 2006). Caring for others requires engagement with the entity that needs care, that is, care-givers mobilize time, efforts and affection to be attentive and respond to the needs of others. When engaging in caring relationships with others, people put themselves in what they care for, thus it becomes part of who they are. As such, this engagement not only shapes the entity cared for, but also the care-giver.

Feminist scholarship has long sought to problematize care by interrogating the social and historical context within which care practices take place. Underlining that care is ‘political, messy and dirty’ (Swallow and Hillman, 2018: 3), feminist scholars emphasize that care practices reflect, but also often come to reinforce, existing regimes of power (Ticktin, 2011). Within and outside the laboratory, studies have drawn attention to invisible and marginalized work, pointing, for example, to the contributions of women scientists (Keller, 1985; Kerr and Garforth, 2016) or ‘invisible technicians’ (Shapin, 1989), who care for the instruments, benches and specimens. Feminist scholars put to the foreground the relevance and epistemic value of care in science and technology, arguing that it is through intimacy, affective relations and sensitive attachment to materials of inquiry that science happens and becomes valuable (Latimer and Gómez, 2019). Puig de la Bellacasa (2011), in this journal, suggests that care is active: It is a doing that helps ‘things get done’, as well as an ethico-political commitment that shapes the way we produce knowledge about the world. Reconsidering Latour’s (2004) conception of ‘matters of concern’, Puig de la Bellacasa argues that attention to ‘matters of care’ can help us shed light on the often taken for granted human labor involved in technoscience. Related to this body of work is Hochschild’s work on emotions (Hochschild, 2002, 2003): She argues that emotions are social expressions of the emotional state of the individual. In particular, she sees emotions as work undertaken by people in order to present themselves in specific ways, and in response to the social norms of a social setting. In other words, emotions are performative and have a deeply social dimension.

Within STS scholarship, some authors have pointed to an ‘affective turn’ (Kerr and Garforth, 2016), accompanied by a growing body of literature examining the care and affective entanglements in clinical practice and laboratory work (e.g. Fitzgerald, 2013; Kalender and Holmberg, 2019; Latimer, 2000; Svendsen et al., 2018; Wetherell, 2012). Of particular relevance to this paper, authors have explored the constitutive role of care practices in knowledge making processes. Friese (2013) discusses how scientists integrate caring for animal models into their preclinical research on the bench, on the basis that better care for animals leads to more translatable research. She shows that care is a ‘constitutive practice in experimental systems’ (p. S131), and argues that it is a potentializing practice that can enable better data quality and clinical usefulness. Along the same lines, Lappé (2018) discusses care as a potentializing and stabilizing practice in research on epigenetics seeking to molecularly trace how experiences of early-life adversity (operationalized as maternal care, neglect and abuse) get ‘under the skin’ and impact gene regulation. She describes how scientists at the bench attend to ‘their’ mice, by producing and measuring certain forms of maternal care, for example, separating pups from their mothers and exploring its effects on phenotypes. Lappé thus points to scientists’ care practices and their awareness of how different forms of care shape the reliability and credibility of epigenetics knowledge itself. What we learn from these different contributions is that care need not be opposed to knowledge, but is constitutive of knowledge making processes, critical to the production of accurate and reliable findings, and forming an integral part of scientific knowledge production.

In this article, we build on and extend these different bodies of literature to shed light upon and problematize the often taken-for-granted human labor that goes into making valuable data within data-intensive research. The notion of care helps us attend to the relational and social dimensions through which data are produced. While we explore the constitutive role of these caring practices for the data, we also examine what these data caring practices do for the staff, the laboratory and the production of knowledge in data-intensive research.

## Research methods and the laboratory

From January to June 2016, the first author (CP) conducted an ethnographic study in an UK-based laboratory carrying out genetics research on twins based on a large database of clinical and research data. For the purpose of this article, we call the laboratory Twinomics. In genetics research, twin studies aim to reveal the importance of environmental and genetic influences for traits, phenotypes and disorders by exploring differences between twins, who share the same DNA. During the time of fieldwork, research at Twinomics was primarily focused on the study of epigenetics, microbiome and transcriptomics, and explored complex diseases with a particular interest in age-related diseases, including osteoporosis, diabetes and cardiovascular diseases. As Twinomics is mostly a ‘dry-lab’, scientists conduct computational or applied mathematical analyses of their twins data and, using an epidemiological approach, look for the incidence and distribution of specific traits in populations.

Work at Twinomics is centred around a registry of data gathered over several decades. This holds data from thousands of twins, with clinical, physiological and lifestyle data, as well as hundreds of phenotypes related to common diseases. Scientists outside of the lab, and outside of the university where Twinomics is located, can access and use the data for their own (academic or commercial) research. The lab holds a number of competitive public and private grants to support its research and infrastructures.

At the time of fieldwork, Twinomics employed around 60 people, half of whom were scientists working in seven teams, and the other half were ‘non-research staff’ supporting research and running the twins database. These included research administrators tasked with liaising with participants to book their clinical visits to the laboratory for sample collection, nurses collecting samples from participants, data assistants in charge of data entry, lab technicians dealing with the collected samples, and staff organizing and managing the sharing of data with other research groups and institutions. So-called non-research staff were therefore heavily involved in producing and managing the data upon which the laboratory’s research was based. Researchers at Twinomics were working across seven teams, each of which worked with (partly different, partly the same) datasets organized around an ‘omic’ technology (e.g. epigenomic; transcriptomics) and managed by a PI. And as we show in the following sections of this paper, scientists were also heavily involved in the production and maintenance of data.

The article draws on observations of laboratory work at Twinomics, including computational simulations, lab meetings, and the production of data and its processing by staff. Fieldwork also involved attendance at conferences and workshops together with members of the lab.

In what follows, we discuss three moments in the journey of data at Twinomics, through which samples originating from research participants are turned into data. They exemplify different forms of care staff perform, as they work to produce, process and render data valuable.

## Relating to research participants and harvesting samples

The production of data at Twinomics starts with ‘twin visits’. These are daylong clinical assessments that twins attend together. They can entail more than twenty tests or sample collections, including the taking of blood pressure, blood and saliva samples, hip, spine or whole-body scans, mole counts and lung function tests. The twins clinic, spread out over three rooms, is located within the hospital of which the laboratory is part. Each room, decorated with posters and promotional material about the twins database, is divided into two spaces: one around a desk and shelves containing paperwork, and on the other end of the room, separated by a curtain, a clinic-like space with the necessary equipment to perform clinical tests. Twin visits are run by research nurses (a women-only team) together with PhD students at Twinomics. Today, a so-called experienced team is in charge of welcoming the twins and seeing them through the different tests and sample collections. It consists of David, a PhD student in the laboratory in his final year, and Annie, a research nurse with more than five years’ experience at Twinomics. The first pair of twins has been scheduled for 9am. It is 8:50 and David and Annie prepare the paperwork and materials needed for their visit. They stand around the desk and grab copies of consent forms and information sheets from the shelves above. Some moments later, the telephone rings. It is the reception informing them that the twin pair they are expecting has arrived. Annie walks out the door and welcomes the pair, calling them by their names. She comes back in with them and points to two chairs where they can sit, on opposite ends of the desk. The twins are identical twin sisters. David and the nurse each sit across a twin (‘their’ twin). As Annie sits down, she says to both twins, ‘You are so young, this is amazing, we don’t get so many young twins.’ She chats with them for a few minutes and asks them a number of questions: whether this is the first time they have come to the lab for a twin visit, how their journey to the clinic was, etc. Annie and David now take the twin sisters through the paperwork. They explain the tests that will take place today, and for each of these tests hand out the relevant consent form and information sheet. The sisters go through the paperwork. After reading one of the forms about the lab using pictures from today’s twin visits, one twin turns to her sister and asks whether she feels comfortable with this. The other says yes. Annie joins in the conversation and adds that the lab wants to use these images only for promotional purposes in order to raise the profile of the twins database. The twins nod and both sign their consent forms. While the twins read and sign the paperwork, David and Annie engage them in light conversation. As one of the sisters explains that they study in different parts of the country, David comments ‘This must have been hard not living and spending time together anymore.’ One of them smiles and says ‘Well, it was also a relief, finally some freedom!’ They all laugh, and the other twin looks at her sister and sticks her tongue out. Now that the paperwork is complete, David and Annie each grab a tube. They ask their twin to spit in the tube and fill it with saliva. Both twins start spitting, and after a few minutes, David’s twin has already filled her tube, when the other twin is about halfway up the tube. Grabbing the tube to close it and put it away, David congratulates his twin, ‘Well done, that’s brilliant!’ Annie joins in the conversation ‘Oh wow, your sister is really quick, well done!’, while the twins look at each other and the ‘quick spitter’ winks at the other. This is the first sample collected today and there will be many others. For each test or sample collected, David and Annie encourage a cheerful competition between the sisters.

As the vignette suggests, the production of data at Twinomics first entails engaging with research participants, their bodies, emotions and identities towards the collection of samples. This constitutes care work, as research nurses and PhD students relate to the twins who visit their lab by engaging in light conversation, listening and answering questions or explaining each test they perform. The work by PhD students and research nurses in twin visits is also care work in that it entails being attentive to participants’ concerns and needs, and responding to those needs, for example, by providing additional information or reassurance to the twin sisters who feel uncomfortable about taking some of the tests. As Viney (2018) shows, biomedical professionals collecting data from twins are not just concerned with data collection as such, but they also manage the ‘extra-clinical identities of twin volunteers’ (Viney, 2018: 157) and offer opportunities for twin pairs to spend time together and interact. The production of data at Twinomics therefore entails a relational dimension in the ways it involves creating and sustaining personal relationships with twins, being attentive to their needs, managing emotions and twinship identities, while exploiting a playful competition and comparison. Producing data also entails caring for participants’ bodies. When collecting samples and taking the twins through tests to measure different bodily parts and organs’ performance (e.g. lung test function; eye test; etc.), staff lay hands on them to position their bodies and perform procedures.

These specific forms of care in twin visits are performed by PhD students together with specialized research nurses. PhD students at Twinomics spend one day a week conducting twin visits, or as they put it, they ‘donate’ this time to the database. PhD students are asked by their supervisors and the laboratory’s management to help out with the twin visits in order ‘do their share’ for the lab and contribute to data production, as they would if they were based in a wet lab and had to spend time at the bench to conduct experiments. We argue that this work has two main functions. First, these caring practices have a service function, that is, by caring for research subjects in twin visits and helping out with data production, staff participate in maintaining it as a functioning research facility. This resonates with Knorr Cetina’s (1999) notion of ‘laboratory caretaking’. In her study of molecular biology’s epistemic culture, she highlights the existence of a series of tasks, activities and roles that are specifically dedicated to the reproduction and maintenance of the laboratory as a facility. This includes, for example, the taking care of workspaces and experimental materials, but also growing cell lines. She explains that every researcher in the molecular biology laboratory studied is involved in carrying out ‘service functions’ that help maintain the laboratory with its materials and equipment. At Twinomics, as staff build relationships with research participants, they enable the collection of samples essential to the production of data, thus providing the materials necessary to build up and maintain the twins database. Through their care practices towards twins to collect samples, staff take part in the collective effort of making and expanding the database.

Second, this specific form of care work is not merely instrumental to the goal of collecting data, but also functions as a rite of passage for PhD students into becoming lab members and constitutes their subjectivity as researchers. By taking part in twin visits, David comes to understand ‘what it takes’ to produce the datasets he works on. More importantly, the caring relationships researchers build with participants in the clinic shape the ways they understand their data and research. While taking part in twin visits and relating to research subjects, researchers experience data production at a bodily and affective level. When David welcomes the twin sisters to the clinic and takes them through the various tests, he listens to their stories about university life and participates in the conversation, sharing his own experiences as a university student, his likes and dislikes about the scientific disciplines taught. When ‘his’ twin fills up her saliva tube quickly, he congratulates her and smiles, while also joking with the research nurse about the competition between the sisters. As such, in his relationships with the twins, David is not the neutral researcher who dispassionately deals with research participants as data donors, but he engages with them as people and brings in his personal experiences and emotions. Through this process of relating and personally engaging with twins visiting the clinic, the data that researchers collect become ‘their data’. They become personally involved and attached to the data they help produce – and the data also retain a ‘face’, the face of the participant. Through this process, the data themselves become relational.

Caring for research subjects in twin visits to collect samples is not regarded as science by members of Twinomics. Instead, this sort of care is understood as a form of ‘housekeeping’ to be undertaken by staff members on the low end of the hierarchy. As Kerr and Garforth (2016) point out, it is often women who take on the housekeeping roles in order to reproduce and maintain the lab. They argue that laboratory housekeeping is marginalized within the laboratory, because it is mostly thought of as bearing a supportive function that enables the more visibly productive forms of work to flourish – work that is formally recognized through scientific credit attribution mechanisms. At Twinomics, we are presented with a more complex picture. On the one hand, we found what Kerr and Garford report: This labor is relegated to the domain of junior research staff or female research nurses, and not rewarded with authorship or acknowledgements in publications. We observed that many of the PhD students at Twinomics were disinclined to take part in visits because it took time away from their ‘proper research work’. As Juliette, a final-year PhD student in the lab, explained, twin visits were not ‘worthy of [students’] time’ because this labor could not be mobilized as tokens of credibility outside the lab. PhD students at Twinomics were concerned with maximizing their future employability by accumulating publications. While the junior research staff showed reluctance to do the invisible work of caring for research subjects, they were still committed to the task, because this was ‘their’ data they were helping to produce, and it held the promise of future publications and epistemic credit. On the other hand, however, we found that the affective and attentive engagements with research participants in twin visits had an additional dimension: It established a lasting link not only between the researcher and the participants, but also between the researcher and the data. Through this process, the data became ‘their’ data and researchers felt responsible and connected to the datasets. As we argue below, these connections, in turn, lead to further investments (of time, effort, and further care) by researchers into the datasets and become a mechanism of value creation for the entire institution.

For PhD students at Twinomics, participating in twin visits is thus an ambiguous kind of labor. It is a form of low status service they have to perform for the laboratory, that takes them away from ‘science’ and the writing of publications; at the same time, this work is part of their training, as they function like apprentices learning ‘what it takes’ to conduct data-intensive research; it is also through this labor relating to the participants that they connect with ‘their’ data and come to value it.

## Valorization through care: Making the database competitive

The laboratory’s database is continually worked on, improved and added to. This work is essential not just to maintain the laboratory as a functioning research facility, but also to make the database unique and attractive for others to use. This ambition is motivated first and foremost by the head of the laboratory, Thor, whose long-term goal ‘is to keep improving the database, so I can keep saying that it’s the best database in the world’. PIs and members of their teams work closely with Thor to achieve this ambition, helping to make the database ‘competitive’ through practices seeking to make the data ever more abundant, versatile and of quality. Researchers work to make the database ‘bigger and better’, and through their efforts, they personally invest in the data and further connect with it. Together, researchers at Twinomics are thus committed to the flourishing and growth of the twins database, and as such, it constitutes a different form of care for data (Hamington, 2004; Ruddick, 1989). In this section, we analyze what this specific form of care fostering the growth of the database entails in terms of concrete practices and division of labor.

One way to make the database competitive as a resource in the biomedical big data community is to make it grow in size and diversity. There are four main ways of making the database grow: first, by adding new twins to the database (through twin visits), second, by creating new variables to expand an existing dataset, third, by adding a new data type, and fourth, by having collaborators using the lab’s data share any changes they make to the data. Variables are added to existing datasets when new information about the twins is collected and stored. For example, the existing DNA methylation dataset could be expanded by adding a new variable that could describe DNA methylation levels in twins according to their diet (e.g. vegetarian, pescatarian, etc.). Adding a new data type means adding an entirely new dataset to the database that would describe the twins on a different bodily level, usually using a different ‘omic’ technology (e.g. epigenomics, microbiome, transcriptomics, etc.). Twinomics’ collaborators can access the lab’s data and use it for their research and this can help the database grow. For example, a research team exploring associations between epigenetics markers and age at menopause can be granted access to the lab’s DNA methylation data together with information about the twins’ menopausal status. As part of their research project, they create a new variable that describes the association between DNA methylation and menopause, which they are then required to share with Twinomics.

During fieldwork, Thor and Daniel, a PI at Twinomics, were considering expanding the database by adding new proteomics data (the large-scale study of proteins). For them, this was worthwhile studying because proteomics data were produced using a new sequencing technology that had not yet been used in large populations or twins. As Daniel explained, this not only meant that ‘there [were] a lot of low hanging fruits’ that could enable researchers at Twinomics to ‘discover something new’ and ‘publish well’ in high impact-factor journals, but also that researchers outside the laboratory would be interested in using Twinomics’ data for their own work. With the addition of proteomics data, Twinomics could gain a competitive advantage in the biomedical big data community, in that they would have data to which no other twin cohort had access. In Thor’s words, proteomics ‘ticked all the criteria of a niche’.

Making the database flourish requires senior members of the lab to be attentive to the ‘needs’ of the database. They review the data currently held in the database to identify what data could be added to it. PIs also mobilize their knowledge of the field to identify new research areas worth exploring and gaining data on. Once a new research area is identified, PIs at Twinomics put together a grant application to fund a new sequencing technology that will analyze the twins’ samples and produce new datasets. When writing the grant application, PIs imagine the future of the database and articulate the potentialities of the newly acquired data. For example, Daniel talks about proteomics data with excitement, arguing that ‘it’s very interesting scientifically [and] it also would allow me to find something before many other people.’ In this process, PIs personally engage with the data, investing emotions and efforts into building its future, but they also want others – grant giving bodies, the research community – to imagine with them the future of the database. They formulate expectations around the data in order to enroll their support and thus enable the fostering of the database (Brown, 2003; Martin et al., 2008). As part of this process of articulating the potentialities of the database and imagining its future, PIs commit themselves to helping the database flourish and grow. Through this specific form of care dedicated to making the database grow, PIs personally connect with the data they help create – it also becomes ‘their’ data. The process is mutually constitutive of the data and the PIs. By putting time, effort and skills into the production of data, the data become part of the PIs’ identities, in the sense of deep relations with it, and personal investments in it.

The care enacted by PIs to foster the growth of the database is accompanied by formal systems of responsibility and accountability. Providing new data to the database grants PIs responsibility for managing it. This entails overseeing the use of the data they helped create, granting access to researchers who wish to use it for their project and controlling how it is used. Here, the extent to which researchers have been personally involved in the data collection becomes an important factor for their investment with the future fate of the database. For example, Mark, the PI of the epigenetics team, was recruited to Twinomics a number of years ago because of his expertise in epigenetics, and DNA methylation more specifically. He was given the responsibility of overseeing the production of DNA methylation data. He mobilized time and effort to add new datasets to the database by applying for funding, while also developing a set of variables to expand the existing DNA methylation datasets. Through this process of making and building the database with DNA methylation data, Mark became personally invested in ‘his’ data and came to care about its future. This involved closely following how the data he helped create were used. For instance, during fieldwork, Olivia and Maria, two postdocs working in the epigenetics team, were starting a research project together exploring environmental influences over DNA methylation. In the meeting room, they sat next to each other and looked at the screen of Maria’s laptop, considering what environmental factor to focus on in their project. Smoking was the first one they discussed and agreed on. After writing it down on her notebook, Olivia noted, ‘we should talk to Mark about our plans’. She explained that Mark ‘owns’ the DNA methylation data and they should ask for his authorization to use it before going any further with their project. Agreeing with Olivia, Maria said ‘once permission is granted, we will proceed’.

Gaining access and permission to use the data from Mark was thus the starting point of any research project within the epigenetics team. In Olivia and Maria’s project, Mark wanted to know about the research questions, the sort of variables they used and whether they would create new ones that could be added to the existing datasets, as well as the analysis plan used. He paid attention to how Olivia and Maria used ‘his’ data in order to best foster the data’s evidential value and thus create epistemic value for the epigenetics team, while he was also concerned with how their work on the data could help ‘his’ datasets grow. With Twinomics as a bioresource, data are also produced in the lab with the aim of being used by numerous actors outside the laboratory. Like the other PIs in the lab, Mark grants most data access requests, and oversees who uses the data and how, suggesting changes in their collaborators’ plans, and requiring them to share any changes made to ‘their’ data. Thus, PIs continue to engage and relate to the data they helped produce, looking to make the data grow and foster its value. The PIs’ personal investment in the data generates value for Twinomics, in that they are willing to go at great length to make sure ‘their’ data are used well, producing valuable research.

With the responsibility for the newly acquired data also comes authorship rights, as PIs are granted senior authorship on publications which use the data they helped produce. A week after their first meeting, Olivia and Maria met again. Mark had now officially authorized the project and granted them permission to use the DNA methylation data. In this second meeting, they began drafting a one-page abstract for the project and agreed on the authors’ list for future publications emerging from this project: Olivia was the first author, Maria the second, while Mark was granted senior authorship. This formal accreditation system at Twinomics rewarded Olivia and Maria, the postdocs, for their work analyzing the data and making knowledge, while it rewarded Mark for his work producing the data. Put differently, at Twinomics, PIs are rewarded for providing the lab the means of production upon which new knowledge claims are made.

To make the database an attractive research resource, data need not just be available in sufficient variety and quantity, but it must also be of good quality. This entails turning the ‘raw’ data received from the sequencing technologies (e.g. the DNA data from twins coming out of the sequencing machine, before it is interpreted) into ‘clean’ and organized datasets ready for analysis. PIs task junior staff on their teams with this work. When asked about a typical day of work, Juliette, a PhD student on the epigenetics team, replied that cleaning the data is an important part of her work:

I need to clean the phenotype we have, and match it with the ‘omics’ data, and the definition of what is ‘clean’ data, in the clinical point of view but also in point of view of the ‘omics’ data.

This is an onerous interpretive process whereby staff make a series of decisions and subjective judgements about what data are good and how data should be transformed. This process draws on the staff’s expertise and experience working with the data (Boyd and Crawford, 2012; Levin and Leonelli, 2017; Neff et al., 2017; Pinel, 2020) while it also requires computational skills. While data cleaning was more often than not seen as a tedious and frustrating task, it was also highly valued. Juliette explains:

When you have to clean your data, you are trying to reduce background noise, which comes from batch effects. … If we don’t do it at the beginning, we will have this noise on the data, then we might make a wrong interpretation. An interpretation can be wrong just because there is some noise.

By cleaning the twins data, researchers transform it into a useful resource, which can be trusted when analyses are done. Junior researchers also mobilize their scientific expertise to label the data and classify it into categories linked to particular keywords to facilitate search and retrieval. In addition, PhD students and postdocs make sure that every set of data is accompanied by metadata, to enable later data users to understand the context in which the data was produced (Edwards et al., 2011). Leonelli (2016) refers to this work as ‘packaging’ and ‘curating’ the data, so that it can be ‘decontextualized’ and travel to different contexts from its origin, to then be ‘recontextualized’ by new data users who understand the context in which the data was first produced. Instead of understanding this work as merely about enabling the travel of data, we argue that it further establishes a relationship between the researcher and the dataset. For example, PhD students who participate in twin visits are already personally invested in the data and continue to connect with it when cleaning and organizing datasets. Feeling responsible for the data they helped produce, they invest further time, effort and affect into making it ‘bigger and better’. Although they see data cleaning and organizing as tedious, they are committed to accomplishing this work because they want to make ‘their’ data grow and enhance its quality. In this sense, this work is similar to many tedious tasks involved in other caring relationships, for example a child assisting an elderly parent getting bathed or dressed. It is a laborious, yet necessary, task the care-giver accomplishes to preserve, foster or protect the care-receiver. At Twinomics, as researchers clean data, they do not show particular enthusiasm. Instead, they often appear frustrated or concerned when, for example, data is ‘missing’ from datasets, but they carry out the task of cleaning the data anyway. It is an emotional and personal investment in the data, which, in turn, strengthens the connectedness between researchers and data. The personal relationships researchers form with the twins data is source of value for Twinomics as researchers are willing to do more for the lab’s data.

This relationship becomes inscribed in the data and in the researchers and contributes to the value of the dataset in that it creates ways of knowing. Researchers *know* their datasets, that is, they have contextual and practical knowledge about what a dataset seeks to represent, what its strengths and weaknesses are, how it should be formatted to become usable. In other words, without data care, data are harder to interpret and use. This form of care seeks to foster the ‘basic capabilities’ (Engster, 2007) of data by turning the unusable raw data into a functioning resource that can be mobilized across contexts and projects to make new claims about the world. This form of care is therefore also essential for Twinomics to generate epistemic value from the data.

While essential to make the database flourish and increase in value, this form of data care enacted by PhD students and postdocs is not rewarded within established valuation metrics and credit attribution mechanisms of the university. Instead, cleaning and organizing data is understood as a service that junior researchers perform for their respective team. Processes and instruments of external valuation such as academic reward systems tend to recognize the care work of PIs who provide data for the lab and the community to use, while it renders invisible the work of PhD students and postdocs performing the tasks of cleaning and organizing associated with the domestic. Without evaluative instruments appropriately taking into account the different data caring practices, we not only ignore the social, relational and contextual dimensions of data-intensive research, but also render invisible the constitutive relationships humans have with data and the fact that these practices are value generators.

## Transforming data into valuable research tools

Olivia and Maria are meeting to discuss their common research project exploring environmental influences over DNA methylation. They walk to the meeting room on the analysts’ floor, bringing their notebooks and laptops. Katherine, a postdoc in the lab, joins them. Maria and Olivia asked Katherine to come along because they want her input on the DNA methylation data and the statistics of this project. Maria explained in an interview:

[Katherine] arrived at the lab with the [DNA methylation] data. She started her PhD with this data and she has been here for six years, so a long time. She knows everything and she also knows how the data have been processed, because she was here when it first arrived, so she knows about the problems they had.

Over the previous six years, Katherine built a personal relationship with the DNA methylation data: She participated in its collection in twin visits, helped clean and organize it, and developed a wealth of experience working with it to produce research results. She is the ‘go-to’ person at Twinomics for anything to do with the DNA methylation data. During this meeting, they discuss the need to normalize the data, how to format the data, which statistical tests to perform, statistical power and the number of twin pairs they should use to run such analysis. Olivia asks Katherine what she feels about using *beta* or *m* values – these are differently formatted datasets which could be used to conduct the analysis. While Olivia mentions *m* values, Katherine shakes her head strongly and interjects, ‘No no, I don’t like *m* values, it doesn’t work in this data. A nice *beta* would work better.’ Katherine bends over the table to take Olivia’s pen and notebook. She draws a number of plots illustrating what the two differently formatted datasets would mean for the data distribution and lead to in terms of results. Katherine also argues that they should normalize their data to conduct the analysis. Normalizing the data means formatting and structuring it in order to enable a certain data distribution (e.g. reducing data redundancy, improving its ‘integrity’). A normalized dataset enables researchers to apply specific statistical tests. Maria is unsure about normalizing, as she feels ‘it transforms the data’. Katherine insists and suggests ‘running the analysis’ on both normalized and non-normalized data. She adds ‘if you don’t run it, you don’t know’. The next day, Olivia is running tests on her computer. She is performing a number of statistical tests on differently arranged datasets. One of these tests finishes and she opens a figure on her screen. She jumps off her seat to dance a few steps and says, ‘Somebody has to give me a reason for not using this test.’ Throughout the afternoon, Olivia alternates between attentively typing in lines of codes, nervously waiting while the scripts are running, and enthusiastically analyzing plots. Her reactions are passionate and each new plot she opens is welcomed with excitement. By the end of the afternoon, she keeps six different plots, after running the analysis on four datasets and using two statistical tests.

In order to produce results, researchers at Twinomics need to be attentive to the specificities of their data. They construct a specific dataset for each of their research projects that is best ‘fitted’ to the questions they ask and the analysis they conduct. They process and prepare the data so as to compose ‘their dataset’. This entails selecting the twins that work best for the study, choosing to normalize or not the data, and formatting it in ways that will make possible the use of statistical tests. Through these practices, researchers foster and develop the data’s ‘capabilities’ to make new claims about the world and produce knowledge. As such, this labor constitutes care because it is a potentializing practice (Friese, 2013) through which researchers hope to produce accurate, reliable and valuable findings.

The relational and deeply personal dimension of this labor is also what makes this work ‘caring’. Preparing and structuring the data for analysis fosters researchers’ personal involvement with the data in order to, as Maria argues, ‘know how the data behave’ and best use it for the production of results. In the example above, Katherine has developed ‘intimate’ knowledge of the data over time through direct interactions with it (Friese, 2019). She knows what the DNA methylation data need in terms of formatting to fit certain analyses (e.g. using *beta* values instead of *m* values). In the meeting with Olivia and Maria, she speaks for ‘her’ data, indicating how it likes to be ‘treated’. When Olivia ‘runs’ a number of tests on the data, she learns about its preferences and ‘behaviors’. This process is far from neutral: As Olivia attentively codes lines to format the data, she puts in effort and has expectations and responds to results with enthusiasm and emotions. As she puts a bit of herself in processing the data, she becomes personally attached and cares for it. At Twinomics, preparing data for analysis is thus a deeply relational and personal labor, from which emotions and affects are not absent. It is through this relational labor that the data exist as valued objects that can be used to make new claims about the world. This account of knowledge-making processes at Twinomics alters the portrayal of scientists dispassionately manipulating their research objects, or the vision of data-intensive research as the mechanical process consisting of researchers ‘running analyses’ of large-scale datasets on their computer. In addition, it shows how Twinomics fosters researchers’ personal relationships and affective engagements with the data as a way to make valuable data and create value.

These practices, through which researchers engage with their data and learn how to best use it in their analyses, are a form of tacit knowledge acquired through doing. As Katherine puts it, ‘if you don’t run it, you don’t know’. Researchers enact a number of formatting techniques on the data at hand, and through this process of engaging with the data, learn how data need to be ‘treated’ to become valuable. This is what Olivia does in her project with Maria, carrying out the analysis on a number of differently arranged datasets ‘to see what happens’. More specifically, she observes that some datasets work better than others for the analysis plan she developed. Learning from this observation, she then applies normalizing techniques and statistical tests that format the data to fit her analysis. Thus, it is in practice that Olivia learns about the needs of the data and how best to respond to them in her analysis. Data caring practices do not simply consist of acquiring knowledge and applying it in action as per linear accounts of practice (Cook and Wagenaar, 2012; Schön, 1983). Instead, knowing and doing are folded into one another (Wagenaar, 2004) in data caring practices: Researchers need to personally engage with the data in practice and put effort and time into this labor to know how to care for it. The work of processing and formatting the data in practice in the context of action also indicates that knowledge making within data-intensive research is a deeply iterative process (Tanweer et al., 2016) rooted in the data and the relationships researchers form with their data during the conduct of their work.

Through these specific practices whereby researchers process and transform the data into a research resource, the data become valuable in different respects for scientists. As researchers personally engage with the data, learning about their preferences and ‘behaviors’, data become valuable on a relational level. The data become personal and meaningful to the researchers because of the emotions, time and skills they invest in it. At the same time, through these caring practices, the data also gain instrumental value within the lab and in the data-intensive scientific community. The formatting and processing of data is critical to the production of accurate and reliable findings. In a similar vein as researchers care for mice in epigenetics of early life in order to stabilize experiments and enable the production of robust results (Lappé, 2018), scientists care for data at Twinomics to produce conditions of possibility for their research. As such, processed data have academic value in that they can lead to the production of research results which are publishable. The work of processing and formatting the data for analysis is highly valued within the lab because it helps scientists achieve results which are ‘trustworthy’, stable and accurate. The academic and evidential value of data is recognized through the evaluative systems of the 21^st^ century university. Researchers who process and format data are granted authorship on publications because they fostered the evidential value of the data.

## Conclusion

Data used in data-intensive research are produced and maintained in relationships with humans who care. Data need to be cared for to become valuable and meaningful to researchers and their work. In our study, care for data starts in the clinic with twins donating samples. Staff begin creating caring relationships with the laboratory’s data by personally engaging with twins and attending to their needs. When relating to twins, staff invest time and affect towards making data for the lab, and through these affective engagements, the data become ‘their’ data. Care for data also happens when adding new data to the database, with PIs writing grant applications. They imagine the future of the database and articulate the potentiality of data in order to foster its growth. They invest in time and effort making the database become ‘bigger and better’. They do so because they are personally involved in the data and feel responsible for it, while caring for data in such a way also enables them to enhance the value of the database. PIs continue caring for the data by overseeing how it is used and by whom. Care for data also happens when cleaning, packaging, processing and formatting the data. Junior staff transform raw data into a valuable research resource that can be used in and out of the laboratory for the production of accurate and reliable findings. In this process, they further connect with ‘their’ data, and invest yet more time, efforts and affects in making the data valuable. These data practices constitute care practices in that they entail personal engagement with the data to learn about its preferences and behaviors in order to foster its potentiality as a research resource. Through these affective and attentive practices data are made meaningful and valuable. Such care practices are mechanisms of value creation for Twinomics. As researchers build relationships with data, they feel connected to the data and responsible for its flourishing and growth, and are thus willing to go at great length to make the data valuable.

Seeing such data practices as care adds important dimensions to the social science analysis of data-intensive research. First, contributing to critical data studies, our analysis makes explicit how data are not finite objects, but they are continually worked on and added to by staff who imagine the future of data and mobilize skills, attention, and efforts to build its potentiality. In particular, our work speaks to scholarship that argues that data are not neutral entities that scientists dispassionately work on and ‘play with’ to produce knowledge. We show that affective and attentive relationships with data become conditions of possibility for ‘good data’. Scientists build long-term caring relationships with ‘their’ data as they help create it and work with it day after day. Second, our analysis adds to critical data studies by pointing out that it is also through care, emotional and attentive affordances and connections with the people involved in their creation that data gain value. Care for data therefore represents an important dimension of data work. In postgenomics research, it is usually assumed that data are highly valuable entities because of the technology, skills and human labor that go into producing them, while they enable the production of knowledge. When thinking about data practices as care practices, we understand that data are also valuable because humans, who produce and sustain them, care. The relations researchers build with data in twin visits by personally engaging with participants or during their work formatting the data and being attentive to its ‘behaviors’ shape how they come to value the data. Through these care relationships, the data gain relational value at the same time that staff’s subjectivities, belonging to the laboratory and research capacities are constituted.

Analyzing data practices through the concept of care helps us problematize data-intensive research and the complex politics within which data are made. Being attentive to ‘matters of care’ (de la Bellacasa, 2011) within the laboratory, we show that data are never raw (Gitelman, 2013) nor neutral, but are produced and made valuable within networks of care embroiled in specific hierarchical arrangements. Different care practices are differently valued by lab members, university management, and formal reward and review systems, with on the one hand, the work of PIs fostering the growth of the database by applying for funding, and on the other hand, junior staff personally engaging with twins and investing time and emotions into formatting the data. Such affective and attentive engagements with data, which are currently not valued and relegated to the domains of junior staff, are in fact value generators for laboratories like Twinomics, and should be recognized as such. It is often because researchers have personal relationships with the data they help produce that they are willing to do more for the laboratory’s data. With these findings, we not only make visible the invisible affective and attentive labor underlying data-intensive research, but we also problematize the neglect of care, while contesting the valuation systems and instruments operating within universities of the 21^st^ century that render such care practices invisible.
